# Research progress in the prevention and treatment of radiation-induced heart disease

**DOI:** 10.3389/fphar.2025.1745302

**Published:** 2025-12-19

**Authors:** Ye Sun, Chenyi Zheng, Lin Li, Shenglin Zhang, Jiajuan Guo, Jincheng Lv

**Affiliations:** 1 Affiliated Hospital of Changchun University of Traditional Chinese Medicine, Changchun, China; 2 China-Japan Union Hospital of Jilin University, Changchun, China

**Keywords:** inflammation, oxidative stress, radiation-induced heart disease, radiotherapy, reactive oxygen species

## Abstract

Radiotherapy (RT) is a cornerstone treatment for thoracic malignancies, but is associated with an increased risk of radiation-induced heart disease (RIHD), a major cause of long-term morbidity and mortality in cancer survivors. Ionizing radiation directly damages cellular components (proteins, lipids, and DNA), disrupts the mitochondrial electron transport chain, and activates enzymes such as NADPH oxidases, this leads to excessive production and accumulation of reactive oxygen species (ROS). Oxidative stress triggers the pro-inflammatory NF-κB pathway, pro-oxidative MAPK branch of IGF-1 signaling, and the pro-fibrotic TGF-β1 pathway. These cascades promote chronic inflammation, endothelial dysfunction, and microvascular damage, leading to myocardial fibrosis and dysfunction. Antioxidant and anti-inflammatory therapies represent a promising approach for the clinical management of RIHD. Preclinical evidence has suggested that statins, angiotensin-converting enzyme inhibitors (ACEIs), and natural antioxidants such as sodium Tanshinone IIA sulfonate mitigate RIHD by scavenging ROS, reducing inflammation, and inhibiting fibrosis. However, further clinical validation of these drugs is required for RIHD. This review highlights the current research status of the known pathophysiological mechanisms of RIHD, and the various treatment strategies used for its prevention and treatment.

## Graphical Abstract

**Figure:**
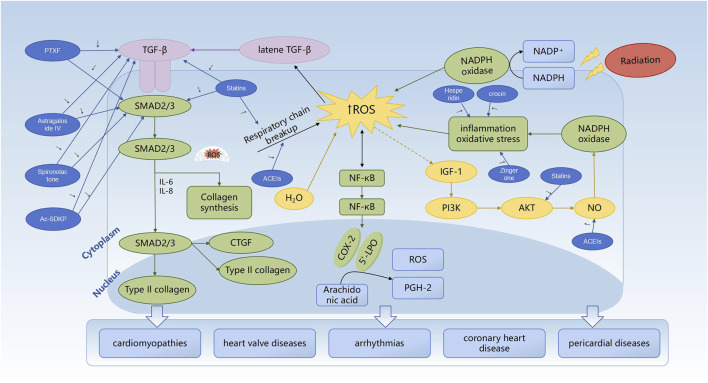

## 1 Introduction

Radiotherapy is a primary treatment for several cancers, especially thoracic solid malignancies such as breast cancer, esophageal cancer, and lung cancer. Advanced radiotherapy techniques such as intensity-modulated radiotherapy, image-guided radiotherapy, and stereotactic body radiotherapy have significantly improved precision, but do not eliminate the irradiation risk for the adjacent organs (Darby et al., 2013). Moreover, beyond planning techniques, specific delivery techniques are crucial in clinical cardioprotection. For instance, in patients with left-sided breast cancer, deep inspiration breath-hold (DIBH) technique increases the distance between the heart and chest wall, significantly reducing the volume and dose of cardiac irradiation, with studies showing it can reduce the mean heart dose (MHD) by approximately 30%–50% (Gaál et al., 2021). Additionally, proton therapy, by virtue of its Bragg peak physical characteristics, can minimize radiation dose to the heart and coronary arteries, making it particularly advantageous for young patients with long life expectancy and for the treatment of mediastinal tumors (Hug, 2018). Radiation-induced heart disease (RIHD) is a serious and dose-dependent complication in cancer patients that undergo radiotherapy, especially breast cancer and Hodgkin’s lymphoma (HL) patients with favorable prognosis. RIHD is clinically manifested as heart valve disease, cardiomyopathy, coronary heart disease, arrhythmia, or pericardial disease. Consequently, it is imperative to increase the awareness of radiotherapy-related risks.

In clinical settings, radiation-induced cardiac toxicity depends on the type and dose of radiation administered (Puukila et al., 2017). Large-scale epidemiological studies have established a clear dose-response relationship for radiation-induced cardiac toxicity. Evidence indicates that for every 1 Gy increase in the mean heart dose (MHD), the long-term risk of major adverse cardiac events (MACE) increases by approximately 7.4% (Darby et al., 2013)Specifically, doses between 1–4 Gy can promote the development of CVDs and inflammation (Weintraub et al., 2010); doses of 5–8 Gy significantly increase the risk of myocardial infarction (MI), angina, pericarditis, and reduced left ventricular diameter; and doses >8 Gy potently induce myocardial fibrosis, a common finding in Hodgkin’s lymphoma (HL) survivors treated with radiotherapy (Russell et al., 2009; Carr et al., 2005; Yusuf et al., 2011). The risk of RIHD is significantly high in patients exposed to radiation doses >30 Gy for one to 2 years. However, the latency period for RIHD may be prolonged in many cases and the disease may manifest decades after exposure to lower radiation doses (Darby et al., 2010). High-dose radiation in cancer treatment damages the cardiac tissue, leading to cardiac dysfunction and CVDs (Puukila et al., 2017). Radiotherapy-induced coronary heart disease represents the second leading cause of morbidity and mortality among breast cancer and HL patients undergoing radiotherapy (Cuomo et al., 2018). The incidence of cardiovascular events is rising among young survivors without traditional risk factors (Raghunathan et al., 2017). Moreover, patients receiving radiotherapy for left-sided breast cancer exhibit significantly higher risk of cardiovascular complications compared to those treated for right-sided breast cancer (Gkantaifi et al., 2019). Despite the immense benefits of radiotherapy, there is a need to limit radiation doses and optimize delivery techniques to limit RIHD (Spetz et al., 2018). Modern radiotherapy techniques have not decreased cardiac toxicity significantly despite reducing cardiac exposure to radiation (Prosnitz and Marks, 2005).

Oxidative stress is the primary contributing factor in RIHD (Taleb et al., 2018) and is caused by an imbalance between reactive oxygen species (ROS) generation and capacity of the antioxidant defense system. Essential cellular components such as lipids, proteins, and DNA are damaged by ROS (Birben et al., 2012). Chronic and acute overproduction of ROS is a significant factor in the development of CVDs (Fuentes et al., 2018). Currently, there is no definitive treatment for effectively preventing the onset and progression of RIHD. In the 2025 Chinese Society of Clinical Oncology (CSCO) Clinical Practice Guidelines for Cardio-Oncology, the Guideline Working Committee of CSCO recommend reducing the extent and dose of cardiac exposure to prevent RIHD. Furthermore, secondary prevention is of critical importance. However, currently, there are no specific secondary prevention drugs in clinical practice to reduce the risk of cardiovascular events following radiotherapy (Oncology GWCotCSoC, 2007). Oxidative stress and inflammation play a key role in RIHD. Furthermore, most chemotherapeutic agents and radiotherapy increase oxidative stress. Consequently, antioxidant therapy is a promising therapeutic strategy to alleviate radiation-induced cardiac toxicity (Wang et al., 2019). Clinical therapies for anthracycline-induced cardiotoxicity such as antioxidants (Hara, 2025) and common cardiovascular drugs (Kalay et al., 2006; Cardinale et al., 2006) exert cardioprotective effects through antioxidant and anti-inflammatory mechanisms. However, whether these drugs can be used to treat radiation-induced cardiotoxicity remains to be verified.

## 2 Molecular mechanisms of radiation-induced heart damage (RIHD): from oxidative stress to activation of signaling pathways

Based on the central role of ROS and the signaling pathways it regulates—NF-κB (inflammation), IGF-1 (oxidative/vasomotor function), and TGF-β1 (fibrosis)—in RIHD, targeting these pathways or their downstream effects represents a promising therapeutic strategy, as discussed in Section 3.

### 2.1 ROS and radiotherapy

Mitochondria are the primary site of oxygen consumption in cells, and the mitochondrial respiratory chain is the main source of ROS (Varga et al., 2015). Radiotherapy disrupts the mitochondrial respiratory chain, leading to reduced ATP production, increased ROS generation, decreased antioxidant capacity, and induction of apoptosis (Vona et al., 2019). NADPH oxidases (NOXs) are the primary enzymatic sources of ROS in the cardiovascular system (Lassègue and Griendling, 2010). NOX2 and NOX4 are the most abundant NOXs in the heart and are primarily expressed in the cardiomyocytes and endothelial cells. These enzymes catalyze the transfer of electrons from NADPH to molecular oxygen, leading to the generation of oxygen free radicals (Seddon et al., 2007). Radiotherapy accelerates ROS levels by activating NADPH oxidase and cyclooxygenase (Azzam et al., 2012). Radiation increases ROS generation and accumulation by altering the upstream sources of ROS and disrupting the balance of endogenous antioxidant defense mechanisms, including glutathione, ascorbic acid, and catalase (Najafi et al., 2017). ROS are highly reactive and damage cellular lipids, proteins, and nucleic acids (Gajowik and Dobrzyńska, 2014). Cardiac muscle cells are rich in mitochondria, with 35%–40% more mitochondria compared to other cells, making them susceptible to damage (Xie et al., 2024).

### 2.2 ROS and NF-κB

ROS activate I-κB kinase, which mediates I-κB phosphorylation, thereby marking it for ubiquitination and proteasomal degradation. This leads to the release of NF-κB, which then translocates to the nucleus, binds to the promoter regions of target genes, and induces the expression of pro-inflammatory cytokines such as TNF-α, IL-1, IL-6, and IL-8 (Morgan and Liu, 2011). Cyclooxygenase (COX-2) and arachidonate-5-lipoxygenase (5-LPO) are NF-κB target genes and are both sources of ROS generation. During arachidonic acid metabolism, both 5-LPO and COX-2 produce prostaglandin H2 (PGH2) and generate ROS. Intracellular ROS also directly activates NF-κB (Morgan and Liu, 2011). A positive feedback loop exists between NF-κB and ROS. ROS activates NF-κB, which in turn increases the production of ROS by increasing the expression of COX-2 and 5-LPO (Rashidi et al., 2017). NF-κB enhances adhesion capacity of the leukocytes by inducing the expression of adhesion molecules. Infiltrating neutrophils exacerbate endothelial cell damage by secreting various pro-inflammatory cytokines. Infiltrating monocytes differentiate into activated macrophages and oxidize low-density lipoproteins (LDLs) via ROS. Subsequently, they transform into foam cells, a process closely associated with the development of atherosclerosis (Ping et al., 2020). NF-κB-induced inflammation is linked with radiation-induced myocardial injury. Increased expression of intercellular adhesion molecules (ICAMs) and vascular cell adhesion molecules (VCAMs) enhances adhesion of the leukocytes to the endothelial cells and thrombi. Subsequently, occlusion of the vascular lumen by microthrombi and vascular stenosis causes filling defects and focal ischemia, leading to myocardial cell death and fibrosis (Slezak et al., 2017).

### 2.3 ROS and IGF-1

Insulin-like growth factor-1 (IGF-1) is the primary mediator of signals from the growth hormone to the body’s tissues (Kenchegowda et al., 2018). IGF-1 also regulates vascular tone by modulating the IRS/PI3K/Akt anti-inflammatory/antioxidant stress pathway and the PI3K-independent pro-inflammatory and oxidative stress pathway involving Grb/Shc/MAPK (King et al., 2016). IRS/PI3K/Akt pathway activation leads to phosphorylation of the endothelial nitric oxide synthase (eNOS) at Ser1179 and the production of nitric oxide (NO). During early stages of radiotherapy, ROS phosphorylates serine 1,177 on eNOS in the human venous endothelial cells, leading to increased NO production (Sakata et al., 2015). ROS react with NO to generate reactive nitrogen species (RNS), thereby reducing the bioavailability of NO. ROS also induce synthesis of vasoconstrictive substances such as prostaglandins and impair vasomotor responses, leading to vascular stenosis (Baselet et al., 2019). IGF-1 stimulates endothelin-1 (ET-1) expression by activating the Shc/Grb/MAPK pathway through IGF-R, IR, or hybrid receptors (Sugamura and Keaney, 2011). ET-1, a potent vasoconstrictive peptide secreted by the endothelial cells, antagonizes the vasodilatory effects of NO, and exhibits pro-oxidative and pro-inflammatory properties (Marquezine and Wajchenberg, 2007). Circulating IGF-1 levels are elevated in minipigs after radiation; moreover, elevated IGF-1 levels post-radiation are positively associated with adverse cardiac events (Kenchegowda et al., 2018). PI3K/Akt pathway inhibition and concurrent activation of the MAPK pathway re-directs IGF-1 signaling towards oxidative and pro-inflammatory responses (Kenchegowda et al., 2018). IGF-1 resistance is associated with vasoconstriction, reduced blood flow, hypoperfusion, and increased oxidative stress, which together form a vicious cycle. It is noteworthy that the tilt of IGF-1 signaling towards the MAPK branch not only directly causes oxidative stress and vascular dysfunction but also can synergize with the TGF-β1 signaling pathway, collectively promoting cardiac fibroblast activation and abnormal extracellular matrix deposition, ultimately exacerbating myocardial fibrosis.

### 2.4 ROS and TGF-β1

Transforming growth factor-β (TGF-β) plays a pivotal role in radiation-induced fibrosis. TGF-β triggers fibrosis through both classical and non-classical signaling pathways. The classical pathway involves activation of target genes such as type I collagen, type III collagen, CTGF, and α-actin by TGF-β via Smad transcription factors (Mollova et al., 2015). TGF-β also promotes via Smad-independent pathways, such as Rho/ROCK pathway (Ge et al., 2017). In animal models of RIHD, reduced cardiac function and myocardial fibrosis are observed within 2–6 months after radiation exposure (Dreyfuss et al., 2021). Radiotherapy-induced ROS is a direct activator of TGF-β1, which upregulates collagen synthesis in a dose-dependent manner (Yarnold and Brotons, 2010). ROS and TGF-β1 form a positive feedforward loop that amplifies fibrotic signals (Ahamed and Laurence, 2017). Recent research reveals that the crosstalk between ROS and TGF-β1 extends beyond direct activation. ROS can induce histone modifications (e.g., H3K27ac) (Randhawa et al., 2023) and DNA demethylation at the TGF-β1 promoter region through epigenetic mechanisms (Oba et al., 2018), thereby maintaining sustained high expression of its pro-fibrotic genes. This epigenetic “reprogramming” may be a crucial molecular memory mechanism underlying the progression of RIHD years or even decades after radiotherapy, also offering new theoretical perspectives for developing epigenetically targeted drugs.

## 3 Potential therapeutic strategies

Numerous studies have confirmed that oxidative stress is the primary mechanism underlying RIHD (Ping et al., 2020). Building upon the central roles of ROS and its regulated NF-κB (inflammation), IGF-1 (oxidation/vasomotor function), and TGF-β1 (fibrosis) signaling pathways in RIHD, pharmacological agents targeting these pathways or their downstream effects have emerged as promising therapeutic strategies. Previous clinical research studies have demonstrated that RIHD can be effectively prevented or treated using antioxidants (Gajowik and Dobrzyńska, 2014), or drugs such as statins (Ostrau et al., 2009), angiotensin-converting enzyme inhibitors (ACEIs) (van de et al., 2015), and metformin (Park et al., 2022).

### 3.1 Statins

Statins are inhibitors of 3-hydroxy-3-methylglutaryl-coenzyme A reductase and are primarily used to lower cholesterol levels. They also demonstrate pleiotropic effects and cardioprotective benefits in preclinical studies (Ziegler et al., 2017; Monceau et al., 2010; Lenarczyk et al., 2015; Kura et al., 2016). In patients who underwent breast-conserving surgery and adjuvant whole-breast radiotherapy (n = 1,481), statin therapy significantly reduces the risk of major adverse cardiac events (MACE) [adjusted HR = 0.34 (95% CI, 0.25–0.44)]. Rosuvastatin and pravastatin demonstrate the highest risk reduction and a dose-response relationship (Huang et al., 2024). Statins also reduce the expression of TGF-β1, Smad3/P-Smad3, ROCK I, and p-Akt in a dose-dependent manner in the Sprague-Dawley rats and alleviate radiation-induced cardiac fibrosis (Zhang et al., 2015). In mouse models, statins exert antioxidant and anti-inflammatory effects and reduce doxorubicin-induced cardiac toxicity by decreasing nitrotyrosine levels, increasing the levels of SOD2, and inhibiting the mitochondrial apoptotic pathway (Riad et al., 2009). Overall, statins protect against RIHD and fibrosis by decreasing lipid levels, inflammation, and oxidative stress.

### 3.2 Angiotensin-converting enzyme inhibitors (ACEIs)

The renin-angiotensin-aldosterone system (RAAS) plays a significant role in cardiac remodeling. ACEIs inhibit ROS production, reduce myocardial damage due to oxidative stress and inflammation, and increase NO production via the bradykinin system, thereby protecting blood vessels (Bertrand, 2004). In rats, treatment with lisinopril after radiotherapy improves left ventricular diastolic function and posterior wall thickness, and increases maximal mitochondrial respiration and reserve capacity (Ortiz de Choudens et al., 2022). Captopril significantly improves respiratory rate and cardiopulmonary density/structure in rats undergoing thoracic radiotherapy. Furthermore, captopril reduces radiation-induced pleural and pericardial effusions and cardiac fibrosis, thereby improving left ventricular end-diastolic pressure (van de et al., 2015). However, only few clinical studies have investigated the cardiovascular protective effects of ACEIs in RIHD. Therefore, further research studies are needed to confirm whether ACEIs can effectively protect against RIHD.

### 3.3 Antioxidants

Oxidative stress and inflammatory responses play critical roles in the development and progression of RIHD. Therefore, antioxidants represent a potential therapeutic approach for alleviating RIHD. Several Chinese herbal extracts and natural compounds alleviate radiation-induced cardiac oxidative and myocardial fibrosis (van de et al., 2015).

Sodium tanshinone IIA sulfonate (STS), a water-soluble derivative of tanshinone IIA, extracted from a traditional Chinese medicinal (TCM) herb *Salvia miltiorrhiza* (Danshen), exhibits several pharmacological effects (Gu et al., 2016; Chen et al., 2017; Xuan et al., 2017). In primary cardiomyocytes (PCMs) from Sprague-Dawley (SD) rats exposed to radiation, STS exerts significant cardiac protective, anti-inflammatory, and antioxidant effects by reducing cTnT leakage, p38/p-p38, and caspase-3 expression levels, and enhances Bcl-2/BAX levels (Ma et al., 2024).

Astragaloside IV (AST), a pharmacologically active component isolated from the Chinese herb *Astragalus membranaceus* (Ren et al., 2023), reduces ROS production in cardiac fibroblasts (CFs) from irradiated rats. AST also decreases the expression levels of Col-1, TGF-β1, and p-Smad2/3 expression and suppresses X-ray-induced downregulation of TIMP1 and Smad7, thereby attenuating radiation-induced fibrotic damage (Gu et al., 2014).

Zingerone, a natural polyphenol (Ahmad et al., 2015) possesses antioxidant (Rajan et al., 2013), anti-inflammatory (Kim et al., 2010), anticancer (Vinothkumar et al., 2014), and antibacterial activities (Kumar et al., 2013). In rats, zingerone pretreatment significantly reduces radiation- or cisplatin-induced cardiac histological abnormalities and cardiac toxicity markers, and increases plasma cardiac troponin T and B-type natriuretic peptide levels. It also significantly decreases oxidative stress by reducing malondialdehyde levels and increases glutathione levels as well as catalase activity; moreover, zingerone reduces inflammation markers in rats (Soliman et al., 2018).

In preclinical studies, pentoxifylline (PTXF; a phosphodiesterase inhibitor) (Samlaska and Winfield, 1994) is effective in decreasing radiation-induced fibrosis, either alone or in combination with α-tocopherol (vitamin E) (Chiao and Lee, 2005). A randomized clinical trial confirmed the efficacy of combined PTXF therapy for radiation-related side effects (Delanian et al., 2003). Dose-dependent cardioprotective effects of PTXF are associated with NF-κB inhibition and downregulation of pro-inflammatory cytokines (TNF-α and IL-6) (Zhang et al., 2005; Ji et al., 2004). PTXF also demonstrates antioxidant properties similar to vitamin E (Delanian et al., 2003) and inhibits TGFβ1 mRNA expression (Liu et al., 2009).

Hesperidin (hesperetin-7-rutinoside) is a bioflavonoid found in plant extracts (e.g., tea and olive oil) and citrus fruits (Tejada et al., 2018; Kilic et al., 2019). Diosmin (HDC), a hesperidin derivative and natural flavonoid (Srinivasan and Pari, 2012) significantly reduces collagen deposition, lipid peroxidation, and malondialdehyde (MDA) levels and enhances SOD activity in rats undergoing radiotherapy, thereby demonstrating anti-inflammatory and antioxidant effects (Koosha and Sheikhzadeh, 2022). The oral administration of 100 mg/kg hesperidin for 7 days in rats scheduled to receive a single 18 Gy dose of γ-ray chest irradiation reduced myocardial oxidative damage, inflammatory response, and fibrosis (Rezaeyan et al., 2016).

### 3.4 Other potential drugs

Apart from statins, ACEIs, and various antioxidants, several other drugs have shown significant potential in preventing and treating RIHD. Recently, aldosterone receptor antagonists have gained increasing attention in treating myocardial hypertrophy and heart failure. The cardioprotective effects of spironolactone are related with the epidermal growth factor receptor (EGFR) (Yavas et al., 2011). In doxorubicin-treated rats, spironolactone (SP) inhibits cardiac toxicity by decreasing TGF-β1 and phosphorylated-Smad3 levels (Liu et al., 2016). Spironolactone also effectively suppresses cardiac fibrosis induced by trastuzumab and radiotherapy in rats (Yavas et al., 2017). Metformin is one of the most effective drugs for treating type 2 diabetes (Bailey and Day, 1989; Inzucchi et al., 2015). Metformin inhibits radiation-induced senescence phenotypes in the human aortic endothelial cells (HAECs) and mice. Metformin increases the expression of DNA repair-related genes (e.g., BARD1 and RAD51) in the senescent and radiation-damaged cells. Therefore, metformin prevents radiation-induced cardiac toxicity by promoting DNA damage repair (Park et al., 2022). N-acetyl-Ser-Asp-Lys-Pro (Ac-SDKP) is a ubiquitous endogenous peptide and an important mediator of the beneficial effects of the ACE inhibitors. Ac-SDKP is the precursor of thymosin-β4 and plays a significant role in tissue healing and cell differentiation (Lenfant et al., 1991; Smart et al., 2007). Ac-SDKP suppresses macrophage-dependent inflammatory and fibrotic pathways by migrating to the perinuclear cytoplasm of rat macrophages and inhibiting the release of the carbohydrate-binding surface protein Mac-2 and reducing TGF-β1, collagen I, and collagen III levels in the irradiated cardiac fibroblasts, thereby counteracting radiation-induced toxicity (Sharma et al., 2018).

## 4 Discussion

Clinical studies have demonstrated that radiotherapy increases the risk of CVDs. Oxidative stress plays a significant role in radiation-induced cardiac toxicity. Oxidative damage to the proteins, lipids, and DNA in the cardiomyocytes and vascular endothelial cells alters multiple signaling pathways, impairs cardiac functions, and ultimately leads to RIHD. Although modern radiotherapy has adopted cardiac-protective techniques to reduce the incidence and severity of cardiovascular complications, the precise mechanisms underlying RIHD pathogenesis are not well understood. The clinical management of RIHD requires a dual emphasis on both prevention and treatment, with prevention taking precedence. Technically, beyond the adoption of IMRT and SBRT, routine implementation of techniques like DIBH and proactive consideration of emerging technologies such as proton therapy for high-risk populations are essential to minimize cardiac exposure at its source. From the perspective of the pathogenesis of RIHD, antioxidant therapy is also an essential treatment to reduce the incidence of RIHD at its source. Antioxidant therapy represents one of the most effective approaches for preventing and treating cardiovascular toxicity induced by chemotherapy, endocrine therapy, and targeted therapy. However, whether antioxidant therapy can be used for the prevention and treatment of cardiovascular toxicity caused by radiotherapy remains to be validated. According to the 2025 edition of the CSCO Guidelines for Practice of Cardio-Oncology, anthracycline-induced myocardial toxicity can be prevented and treated using statins, ACE inhibitors (ACEIs), antioxidants, and drugs such as crocin because of their antioxidant effects (Motlagh et al., 2021; Su et al., 2021). These drugs mitigate chemotherapy- or radiation-induced cardiac injury by reducing oxidative stress and inflammation. Although these agents attenuate chemotherapy-related myocardial damage and alleviate RIHD, it is still not clear whether they represent the optimal therapeutic approach for RIHD management. At the same time, these drugs’ clinical translation faces challenges regarding the level of evidence. Currently, the majority of supporting evidence comes from animal models or small-scale, retrospective clinical analyses, lacking confirmation from large-scale, prospective, randomized controlled trials (RCTs). Future research must address several key issues: (Darby et al., 2013): determining the optimal treatment window (e.g., initiating prophylactic administration concurrently with radiotherapy or intervening after subclinical injury occurs); (Gaál et al., 2021); establishing standardized drug dosages and treatment courses; (Hug, 2018); evaluating the safety of long-term medication use, particularly the potential “double-edged sword” effects of certain antioxidants. Specifically, some antioxidants can act as oxidants under certain conditions and exacerbate lipid peroxidation or induce DNA damage (Pérez-Torres et al., 2017). For example, hesperidin functions as a pro-oxidant and modulates hepatic fatty acid oxidation in rats (Constantin et al., 2013); and (Puukila et al., 2017) identifying dominant populations that may benefit from specific interventions through biomarkers. Another key to overcoming the clinical translation bottleneck lies in the early diagnosis and risk stratification of RIHD. Identifying sensitive and specific biomarkers is crucial. Currently, high-sensitivity cardiac troponin (hs-cTnI/T) can detect myocardial microdamage caused by radiotherapy during the subclinical stage (Cirnigliaro et al., 2023), while B-type natriuretic peptide (BNP/NT-proBNP) is also a potential biomarker to heart damage (Zhang et al., 2019; Thavendiranathan et al., 2023). The combined monitoring of both shows promise for identifying high-risk patients and guiding the timing of initiating prophylactic medication. Furthermore, advanced cardiac imaging techniques, such as T1 mapping and extracellular volume (ECV) fraction measured by cardiac magnetic resonance, can non-invasively and quantitatively assess early myocardial fibrosis (Mukai-Yatagai et al., 2018), providing potential objective imaging biomarkers for evaluating the efficacy of interventional drugs and serving as surrogate endpoints in clinical trials. Future efforts against RIHD require a multifaceted approach, combining precision radiotherapy, cardioprotective drugs, and robust biomarkers, while further elucidating underlying mechanisms to improve patient outcomes.
